# Allergy blog: health information

**DOI:** 10.1186/1939-4551-8-S1-A136

**Published:** 2015-04-08

**Authors:** Maria De Fatima Emerson, Neide Maria Pereira, José Leonardo Sardenberg, Katiuscia Almeida Albuquerque, Maria Carolina Costa, Carla Leal Seifert, Matheus Henrique Carvalho Ribeiro, João Bosco De Magalhães Rios

**Affiliations:** 1 Policlinica Geral Do Rio De Janeiro, Brazil; 2 Policlinica Geral Do Rio De Janeiro - Faculdade Medicina De Petrópolis, Brazil

## Background

The Allergy Blog (http://www.blogdalergia.com) was created in 2006 by Allergists at the *Policlinica of Rio de Janeiro* in Brazil with the goal to provide quality information on subjects related to allergies, understanding diseases, forms of prevention, treatment and other practical tips for patients, families and caregivers alike. Currently, there are over five million hits on the site making it a popular tool for medical information seekers. The site also encourages interactive participation for its visitors and has registered over 12,000 emails and 16,000 comments published to date. The purpose of this study is to assess the information and comments published on the site and to understand the interest and needs of the public who engage with the Allergy Blog.

## Methods

The comments received on the Allergy Blog are reviewed and monitored by a moderator for publication. Posts whose contents contain information pertaining to specific treatments, are deemed as ‘miracles’ or, if they are deemed offensive for publication, are rejected. To this date there have been over five thousand comments published and analyzed based on the following topics and include requests for clarification and understanding of: Types of allergies; Types of medicine; Illnesses and preventive measures; Immunotherapy, tests and procedures; Medical diagnosis; Advice or ‘second opinions’ and Medical treatment procedures.

## Results

Over five thousand reviews have been received and analyzed offering the following information for data requests: 22.73% (1137) treatment requests; 22.57% (1129) diagnostic applications; 18.69% (935) information about diseases; 16.33% (817) drug information; 7.64% (382) medical advice and a ‘second opinion’; 6.80% (340) immunotherapy tests and procedures. The requests that were least received were those of prevention measures.

## Conclusions

The Allergy Blog is an innovative online information resource that has provided ethical and concise material for patients about disease prevention and treatment for over seven years. This study shows that the online visitors contact the Allergy Blog primarily for information regarding medical treatment and diagnosis and less for information about preventative measures for illnesses. This study also identifies the significant number of online searches for medical advice and those seeking a ‘second opinion’ for patient diagnosis and treatments.

